# Combined reconstruction of complex chest wall defects using mesh-supported rigid materials and abdominal flap strategies: a retrospective clinical study

**DOI:** 10.3389/fsurg.2026.1821986

**Published:** 2026-05-21

**Authors:** Bocheng Zhang, Ying Long, Bo Song, Xiao Zhou, Zan Li, Bo Zhou

**Affiliations:** 1 Department of Breast Oncoplastic Surgery, the Affiliated Cancer Hospital of Xiangya School of Medicine/Hunan Cancer Hospital, Central South University, Changsha, China; 2 Translational Medicine Center, the Affiliated Cancer Hospital of Xiangya School of Medicine/Hunan Cancer Hospital, Central South University, Changsha, China

**Keywords:** chest wall reconstruction, DIEP flap, mesh-reinforced prosthesis, oncoplastic surgery, radiation necrosis, TRAM flap

## Abstract

**Background:**

Management of recurrent or primary chest wall tumors and radiation-induced ulcers poses significant challenges due to extensive full-thickness defects following radical resection. Simultaneous reconstruction of the thoracic skeleton and soft tissue is imperative for preserving chest wall functionality. This study addresses this clinical dilemma through optimized surgical strategies.

**Methods:**

A retrospective cohort analysis (2016-2023) included 42 consecutive patients with Mathes-Salibian grade III/IV defects at Hunan Cancer Hospital. Outcomes were assessed for bony reconstruction (titanium alloy/polypropylene mesh vs. bone cement/mesh) and soft-tissue coverage (free/pedicled flap combinations).

**Results:**

Among 42 patients (41 female, 1 male), 38 presented with full-thickness defects. Bony reconstruction utilized titanium alloy/nylon mesh (*n* = 7) or bone cement/nylon mesh (*n* = 27). All cases required flaps, with 28 patients receiving combined free deep inferior epigastric perforator (DIEP) flaps and contralateral pedicled transverse rectus abdominis myocutaneous (TRAM) flaps. Postoperatively, one mortality occurred due to multi-organ failure; local complications were managed conservatively. During mean follow-up (30.29 months), 2 patients succumbed to metastatic breast cancer and 1 to cardiovascular events. Remaining patients showed no ulcer recurrence or local tumor progression.

**Conclusion:**

Mesh-reinforced rigid reconstruction provides stable thoracic restoration. The integrated DIEP-TRAM flap approach delivers extensive vascularized coverage with 97.7% successful defect resolution, establishing an effective protocol for complex chest wall rehabilitation.

## 1 Introduction

The chest wall is a dynamic composite structure critical for respiratory mechanical stability, with its three-layered architecture (osseous framework-musculodynamic system-cutaneous covering) ensuring intrathoracic negative pressure. Defects exceeding 5 cm × 5 cm cause paradoxical breathing, reducing vital capacity by over 40 % (1). In oncology, improved breast cancer survival has increased radiation-induced chest wall ulcers (RICWUs) to 12%–15%. Radiation-induced tissue damage progresses via post-radiotherapy microangiopathy, lowering tissue oxygen partial pressure below 10 mmHg and reducing fibroblast proliferation by over 60 % (2). Additionally, radical resection for extensive tumor invasion, while balancing function and aesthetics, makes such defect reconstruction a major plastic surgery challenge.

Traditional reconstruction has limitations: the gold-standard latissimus dorsi myocutaneous flap has a 34.8% post-radiotherapy thoracodorsal artery occlusion rate (3). omental transposition suits infected wounds but cannot withstand respiratory shear forces (4). synthetic implants for osseous defects face key challenges in biomaterial-bone integration, hypoperfusion-related poor anti-infective capacity, and respiratory motion-induced mechanical stress.

Biomaterial-bone integration determines implant success. Bioabsorbable bone substitutes mitigate inflammation and osteonecrosis but need optimized mechanical strength and degradation kinetics (5), while synthetic hydroxyapatite (HA) shows superior osteogenic and osseointegration properties (6). Hypoperfusion-induced poor anti-infective capacity is another critical issue; advanced antimicrobial biomaterials, via therapeutic release and surface modification (7), such as vancomycin-loaded hydrogels combined with micro-arc-oxidized 3D-printed porous Ti6Al4 V implants, have shown excellent antibacterial and osteoinductive effects *in vitro* and *in vivo* (8).

Respiratory motion threatens implant stability; bifunctional biomaterials integrating osteogenic and antimicrobial properties enable simultaneous bone regeneration and infection control in contaminated environments (9). Nanomaterials and nanopharmaceuticals reduce multiple surgeries and high-dose antibiotics, shortening recovery and facilitating clinical translation.

This study innovatively adopts a bipedicled abdomino-thoracic transfer (BAT) strategy, using cross-regional hemodynamic integration to provide “biological armor” for titanium alloy/bone cement implants, with a 3D biomechanical design unifying static support and dynamic function. Analyzing 42 Mathes-Salibian grade III/IV chest wall defect cases, we focus on: (1) radiotherapy-adapted bone repair material selection; (2) hemodynamic advantages of bipedicled abdominal flap transfer; (3) clinical pathways for complication prevention and management.

## 2 Methods

### 2.1 Study populations

**Figure 1:**
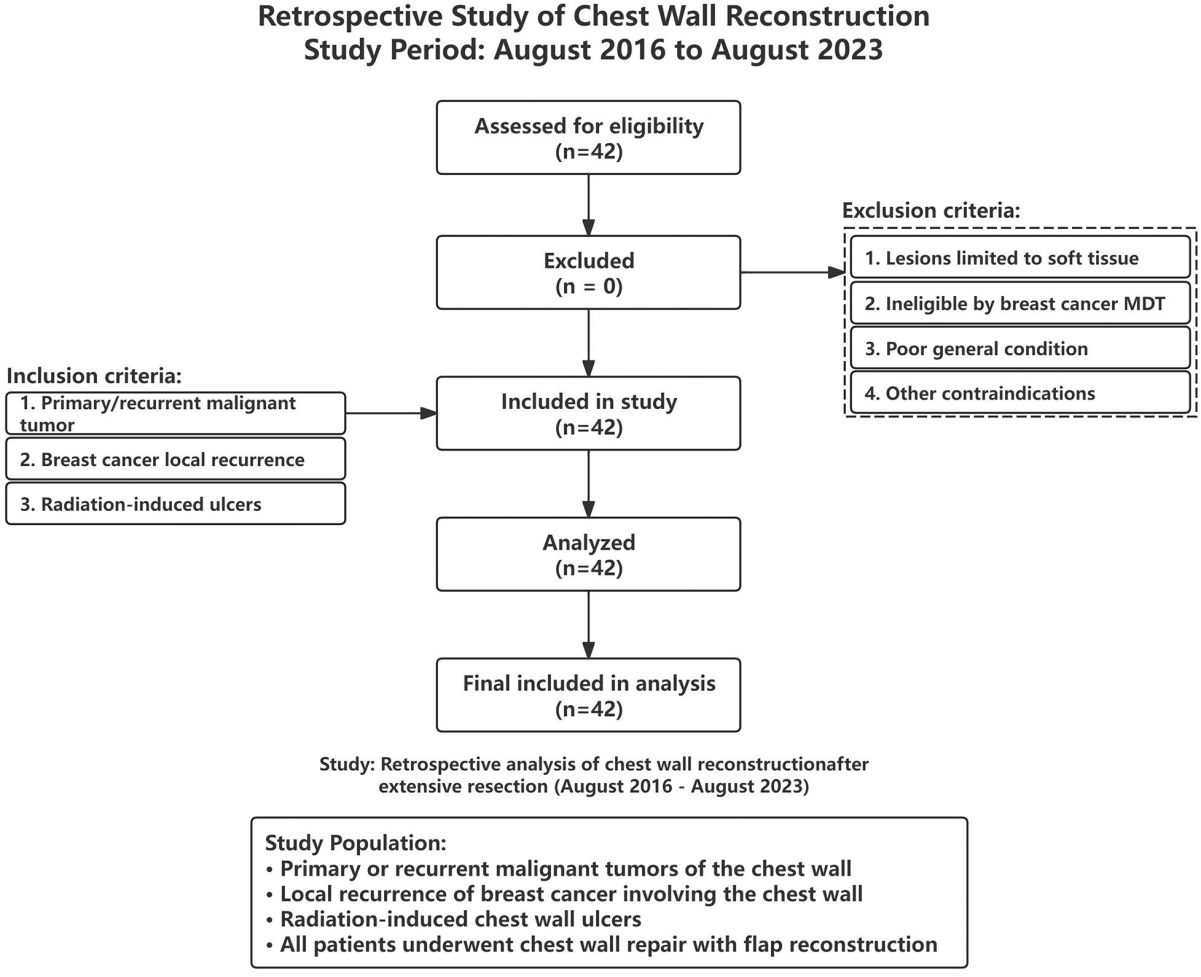
Consort flow diagram for a retrospective study of chest wall reconstruction.

This study conducted a retrospective analysis of chest wall repair cases managed in our department from August 2016 to August 2023. The inclusion criteria for cases were as follows: (1) patients presenting with primary or recurrent malignant tumors originating from the osseous or soft tissue structures of the chest wall; (2) patients exhibiting local recurrence of breast cancer at the surgical site or primary lesions involving the chest wall; and (3) patients with radiation-induced ulcers on the chest wall. The exclusion criteria were: (1) patients whose lesions were confined to soft tissues and amenable to direct suturing without the necessity for flap reconstruction following extensive resection; (2) patients deemed unsuitable for surgical intervention by the Breast Cancer Multidisciplinary Team; (3) patients in poor general health who could not tolerate general anesthesia; and (4) patients with other contraindications to surgical procedures (Figure 1).

**Figure 2.**
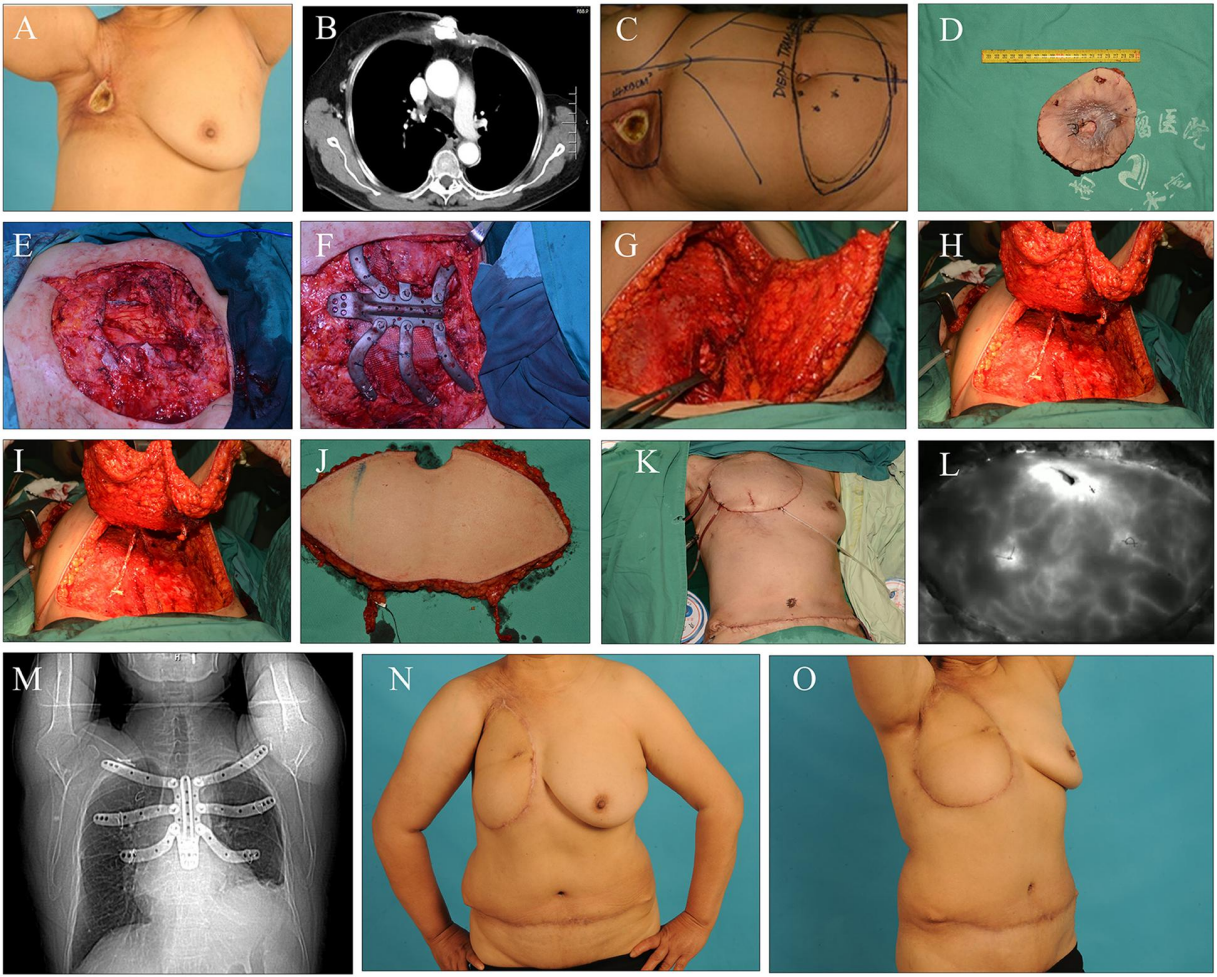
A typical case of complex chest wall defect repair and reconstruction. **(A)**. Preoperative condition of chest wall radiation ulcer **(B)**. Preoperative CT of chest wall radiation ulcer **(C)**. Determination of surgical resection range and flap design **(D)**. Resected lesion specimen **(E–F)**. Full-thickness chest wall defect after lesion resection: repair of thoracic osseous defect using bone cement combined with mesh **(G–J)**. Preparation of the right deep inferior epigastric artery perforator (DIEP) flap **(K)**. Immediate postoperative condition **(L)**. Evaluation of DIEP flap blood supply by indocyanine green (ICG) angiography **(M)**. Immediate postoperative X-ray **(N)**. Postoperative follow-up at 1 month **(O)**. Postoperative follow-up at 2 years.

Based on the established inclusion and exclusion criteria, this study enrolled a total of 42 patients requiring chest wall reconstruction following extensive resection of chest wall lesions. Of these patients, 41 (97.62%) were female and 1 (2.38%) was male, with ages ranging from 39 to 77 years and a mean age of 58.2 years. Lesions were located in the anterior chest wall in 30 patients (71.43%), with sternal involvement observed in 8 patients (19.05%). Additionally, 12 patients (28.57%) had lesions in the lateral chest wall, with axillary involvement in 8 patients (19.05%). Preoperative biopsy and postoperative pathological analyses revealed that 35 patients (83.33%) had radiation-induced ulcers on the chest wall following breast cancer surgery, 5 patients (11.9%) experienced chest wall recurrence post-breast cancer surgery, 1 patient (2.38%) had radiation-associated sarcoma of the chest wall, and 1 patient (2.38%) was diagnosed with a spindle cell malignant tumor (Table 1).

**Table 1:** Case characteristics.

Case characteristics	General information of patients	
**Total number of cases**		**42 cases**
**Age (years)**	Average age (age range)	58.2 (39-77) years old
**Gender composition**	Female/Male	41 (97.62%)/1 (2.38%)
**Pathological characteristics**		**Total cases: 42**
Radiation ulcer		35 (83.33%)
Breast cancer recurrence on the chest wall		5 (11.9%)
Radiation-related sarcoma		1 (2.38%)
Spindle cell malignancy		1 (2.38%)
**Lesion location**		**Total cases: 42**
Anterior chest wall		30 (71.43%)
Lateral chest wall		12 (28.57%)

### 2.2 Surgical method

All patients with chest wall lesions underwent lesion biopsy upon admission, and surgery was performed after pathological confirmation. Intraoperatively, a sterile transparent grid membrane was used to measure the area of the chest wall defect. The maximum length and width of the defect were recorded, and the area was calculated as length  ×  width (cm^2^). For irregular defects, the contour was traced on the membrane and then converted to an equivalent rectangular area for standardization. Comprehensive imaging assessments were performed, including routine enhanced chest wall CT with three-dimensional reconstruction to determine the layers and extent of lesion involvement; computed tomography angiography (CTA) of the inferior epigastric artery to evaluate perforating branches; and other routine preoperative examinations including abdominal color Doppler ultrasound, electrocardiography, cardiopulmonary function tests, and head CT to rule out distant metastasis and surgical contraindications. All patients received multidisciplinary team (MDT) consultation preoperatively, with participating departments including radiology, breast medicine, thoracic surgery, breast oncoplastic surgery, orthopedics, pathology, anesthesiology, and intensive care unit (ICU). The department of radiology defined the extent of involvement, and the department of thoracic surgery delineated the resection area. For patients with involvement of the chest wall bony structures, lung, or mediastinum, the corresponding ribs and lung tissue were resected, resulting in a full-thickness chest wall defect. The departments of thoracic surgery, orthopedics, and breast oncoplastic surgery jointly developed a plan for bony thoracic cage reconstruction, while the department of breast oncoplastic surgery was responsible for repairing soft tissue defects. The departments of anesthesiology and ICU ensured the smooth progress of surgery and postoperative recovery monitoring, and the department of breast medicine guided subsequent patient treatment.

The individualized repair plans adhere to two primary principles: repair of the bony thorax and repair of the soft tissue. The necessity for bony thorax reconstruction is contingent upon the size, location, and number of ribs excised. Based on prior experience, bony thorax reconstruction is typically unnecessary if the thoracic defect diameter is less than 5 cm, or if the defect is situated at the apex of the chest wall or beneath the scapula. Conversely, reconstruction becomes imperative when multiple ribs (≥4) or a portion of the sternum are removed, particularly if the defect is located anteriorly or laterally on the chest wall, thereby significantly compromising thoracic stability (1, 10). Techniques for reconstructing the bony structure include the use of bone cement combined with nylon mesh and 3D-printed titanium alloy combined with nylon mesh. Other considerations for the repair strategy: 1. Subject to healthcare policies, for patients with limited financial resources, a bone defect diameter of >5 cm and <10 cm, and no stable fixation conditions around the defect, we generally prefer to use bone cement combined with nylon mesh to repair the bony defect of the chest wall. 2. For patients with sufficient financial means who can afford self-pay expenses, or those with combined sternal and multiple rib defects where bone cement alone cannot provide a stable structure, titanium alloy combined with mesh is required to repair the bony defect of the chest wall. For patients with multiple rib defects on one side, with or without partial sternal defects, bone cement combined with mesh repair is recommended. In contrast, patients with bilateral rib defects and complete sternal defects are best served by employing 3D-printed titanium alloy combined with mesh as the optimal repair strategy (11). In terms of flap selection strategy, for larger defects, priority is given to flaps with dual blood supply systems, such as bipedicled DIEP flaps or combined flaps with one free and one pedicled flap. Particularly for patients with poor recipient vessel conditions, the combination of TRAM and DIEP is preferred.

In this study, all patients underwent extended resection of chest wall defects, necessitating flap repair due to the inability to achieve direct wound closure. In the context of breast reconstruction surgery, the latissimus dorsi flap is frequently utilized, particularly when vascularized skin and soft tissue are required to enhance the stability and aesthetic outcome of the reconstruction. Empirical evidence indicates that the latissimus dorsi flap significantly enhances the anterior projection and contraction of the lower pole/inframammary fold of the breast (12). Furthermore, the integration of the latissimus dorsi flap with fat transplantation facilitates complete autologous breast reconstruction without the need for implants, a method that has been demonstrated to be both safe and reliable (13). Nevertheless, the majority of patients are post-radiotherapy individuals following breast cancer surgery, resulting in fibrotic local soft tissue that is unsuitable for nearby flap transfer. Additionally, when the axillary region has been subjected to radiation therapy, the anatomical dissection of the thoracodorsal vessels becomes challenging, thereby complicating the application of the latissimus dorsi flap. The abdominal flap has emerged as a more viable alternative due to several factors: it remains unaffected by the axillary radiation dose, possesses relatively straightforward vascular anatomy, and is comparatively easy to operate. Consequently, in this context, the abdominal flap is regarded as the primary choice for reconstruction (14).

## 3 Results

### 3.1 Repair method

In this study, out of 42 patients, 38 (90.48%) presented with lesions affecting the full thickness of the chest wall, while 4 (9.52%) exhibited lesions confined to the soft tissue. Of those with full-thickness chest wall lesions, 34 patients (80.95%) required bony reconstruction, whereas 4 (9.52%) underwent repair using only nylon mesh. Fifteen patients (39.47%) had chest defect areas measuring ≥100 cm^2^, and another 15 (39.47%) had defect areas <100 cm^2^. A total of 23 cases (60.53%) involved the removal of two or more ribs; 27 cases (71.05%) involved the removal of multiple ribs (≥3), 7 cases (18.42%) included rib removal with an accompanying sternal defect, and 4 cases (10.53%) involved the removal of ≤2 ribs without a sternal defect. Among the 38 patients requiring thoracic repair, 7 cases (18.42%) utilized a combination of titanium alloy and nylon mesh, while 27 cases (71.05%) employed bone cement in conjunction with nylon mesh (refer to Table 2). All 42 patients required flap repair, with the most prevalent method being the combination of free flap and pedicled flap, utilized in 28 cases (66.67%). This included the use of a free deep inferior epigastric artery perforator (DIEP) flap combined with a contralateral transverse pedicled rectus abdominis myocutaneous flap (Table 3).

**Table 2:** Repair of chest defects.

Thoracic defect repair	Reconstruction of bony thoracic
**Types of chest defects**	**Total cases: 42**
Perforation defect	38 (90.48%)
Soft tissue loss only	4 (9.52%)
**Perforation defect**	**Total number of cases: 38**
Bony thoracic reconstruction required	34 (89.47%)
No bony thoracic reconstruction required	4 (10.53%)
**Thoracic defect area**	**Total number of cases: 38**
<100cm2	23 (60.53%)
≥100cm2	15 (39.47%)
**Perforation defect**	**Total number of cases: 38**
Multiple rib resection (≥3 ribs)	27 (71.05%)
Rib resection with sternal defect	7 (18.42%)
≤2 ribs removed without sternal defect	4 (10.53%)
**Thoracic repair method**	**Total number of cases: 38**
Titanium alloy combined with nylon mesh	7 (18.42%)
Bone cement combined with nylon mesh	27 (71.05%)
Pure nylon mesh	4 (10.53%)

**Table 3:** Soft tissue repair.

Soft tissue defect repair	Reconstruction of soft tissue
**Flap Type**	**Total cases: 42**
Simple free flap	9 (21.43%)
Free flap combined with pedicled flap	28 (66.67%)
Pedicled flap	4 (9.52%)
Adjacent tissue flap (limb flap after upper arm amputation)	1 (2.38%)
**Simple free flap type**	**Total number of cases 9**
DIEP	5
Bilateral free TRAM or free TRAM combined with DIEP	2
Bilateral anterolateral thigh flap	1
Bilateral deep femoral artery perforator flap	1
**Free flap combined with pedicled flap type**	**Total number of cases: 28**
DIEP combined with TRAM	27
Free TRAM combined with contralateral pedicled TRAM	1
**Types of pedicled flaps**	**Total number of cases 4**
Double-sided TRAM	2
Unilateral TRAM combined with latissimus dorsi	1
Simple latissimus dorsi	1

DIEP, Inferior epigastric artery perforator flap; TRAM, transverse rectus abdominis muscle flap.

### 3.2 Typical case

A 53-year-old female patient was admitted to the hospital due to a three-year history of right chest wall ulceration, occurring nine years following comprehensive treatment for right breast cancer. Physical examination indicated alterations in the right chest wall consistent with post-radiotherapy changes. An ulcerative lesion, approximately 6 cm by 5 cm in size, was observed near the axilla in the subclavian region, characterized by gray-white necrotic tissue and minimal secretion. Preoperative biopsy of the ulcerative lesion revealed no evidence of malignancy. Chest computed tomography (CT) demonstrated slight thickening of the soft tissue in the right chest wall and a small amount of encapsulated effusion in the surgical area, along with radiation-induced pneumonia in the right upper lung. Additionally, abnormal changes in multiple ribs on the right side were noted, suggestive of radiation osteomyelitis. Following comprehensive preoperative evaluations to rule out breast cancer recurrence, distant metastasis, and other surgical contraindications, surgical intervention was planned. After MDT discussion, the surgical plan was determined as follows: extensive resection of the chest wall soft tissue lesion and involved ribs, repair of the bony thoracic defect using bone cement combined with mesh, and reconstruction of the soft tissue defect with a free deep inferior epigastric perforator (DIEP) flap plus a pedicled transverse rectus abdominis myocutaneous (TRAM) flap.

The DIEP flap was meticulously harvested with the following standardized surgical technique: With the patient in supine position, Doppler ultrasound was used preoperatively to mark the dominant perforators of the deep inferior epigastric artery. A transverse abdominal skin paddle was designed according to the size and shape of the chest wall defect. The skin and subcutaneous tissue were incised along the marked outline. The dissection proceeded from the lateral edge toward the medial side, identifying and preserving the reliable musculocutaneous perforators penetrating the anterior rectus sheath and rectus abdominis muscle. Under loupe magnification, each selected perforator was carefully dissected through the muscle fibers, splitting the rectus abdominis minimally to preserve the muscle and its innervation, thus minimizing donor-site morbidity and maintaining abdominal wall function. The perforator was traced proximally to the deep inferior epigastric artery and vein, ensuring a sufficient vascular pedicle length and diameter for microvascular anastomosis. The flap was completely separated from the abdominal wall while maintaining perfusion through the isolated perforator. The vascular pedicle was divided at a suitable length, and the free DIEP flap was transferred to the chest wall defect. Microvascular anastomosis was performed under an operating microscope: the deep inferior epigastric artery was anastomosed end-to-side to the internal mammary artery or thoracodorsal artery, and the concomitant vein was anastomosed to the corresponding recipient vein, with patency confirmed by intraoperative Doppler and indocyanine green (ICG) angiography. All flaps survived completely postoperatively. At the 2-year follow-up, there was no ulcer recurrence, and both the aesthetic appearance of the chest wall and respiratory function were satisfactory (Figure 1).

### 3.3 Complications and follow-up

Following surgery, one patient (2.38%) succumbed to multiple organ failure. Complications were observed in 21 patients, with the most prevalent being poor wound healing in the repair area, occurring in 13 cases (30.95%). Additional complications included postoperative hypoxemia, respiratory insufficiency, atelectasis, and pleural effusion in 5 cases (11.9%), poor healing due to fat liquefaction in the abdominal donor area in 2 cases (4.76%), and a vascular crisis in the free flap in 1 case (2.38%). The duration of postoperative hospitalization ranged from 8 to 61 days, with an average of 17.93 days. The follow-up period extended from 3 to 84 months, averaging 30.29 months. During follow-up, one patient died from brain metastasis of breast cancer 7 months post-surgery, another from multiple systemic metastases of breast cancer 13 months post-surgery, and a third from a cardiovascular accident 22 months post-surgery. The remaining patients survived without experiencing local ulcers or tumor recurrence (Table 4).

**Table 4:** Complications.

Complication	Total number of cases: 22
Die	1 (2.38%)
Complications related to lung function	5 (11.9%)
Poor healing of the recipient site	13 (30.95%)
Donor fat liquefaction	2 (4.76%)
Flap vascular crisis	1 (2.38%)

## 4 Discussion

The four predominant etiologies of chest wall defects include primary or recurrent chest wall tumors, radiation-induced damage, trauma, and infection (15). Clinically, tumors and radiation-induced damage constitute the majority of cases. The chest wall serves several critical functions, such as protecting thoracic organs, maintaining respiratory mechanics, and facilitating upper limb movement. Compromise of chest wall integrity significantly impairs these functions, particularly in cases of recurrent breast cancer or malignant tumors affecting the soft tissue and bone of the chest wall. These conditions often necessitate extensive resections, leading to full-thickness chest wall defects. Substantial defects in the bony structure of the chest wall can result in impaired respiratory mechanics and severely impact both respiratory and circulatory functions (16, 17). The approach to bony chest wall repair is contingent upon the defect's location, size, and the number of ribs excised. According to Deschamps’ findings, chest wall defects with a diameter less than 5 cm typically do not require bony reconstruction. Similarly, defects situated near the apex of the chest, beneath the scapula, or adjacent to the spine generally do not necessitate bony repair (18). However, the anterior and lateral chest walls require further consideration.

The reconstruction of the bony thorax should be tailored to the individual patient, with the choice of repair method contingent upon the defect's location and extent. Presently, the predominant techniques for repairing bony structures include the use of bone cement in conjunction with mesh, as well as 3D-printed titanium alloy combined with mesh. Bone cement, primarily composed of polymethyl methacrylate, offers advantages such as cost-effectiveness, widespread availability, and ease of intraoperative shaping. However, it also presents drawbacks, including biological toxicity, challenges in intraoperative fixation, inadequate postoperative strength, potential for fracture, and postoperative pain and effusion (19). For cases not involving the entire sternum or multiple bilateral rib defects, bone cement combined with mesh remains the most frequently employed method for bony thorax repair. In this study, 27 patients (71.05%) underwent this repair method, with no instances of bone cement fracture, severe pain, or effusion observed during follow-up. Palmesano et al. reported that after chest wall sarcoma resection, the use of polypropylene mesh combined with methyl methacrylate to reconstruct the bony structure and latissimus dorsi flap to cover soft tissues is safe, effective, low-cost, and can prevent paradoxical breathing. This is consistent with the bone cement plus mesh repair strategy in this study, both of which can effectively maintain chest wall stability and improve respiratory function (20). Spicer et al. demonstrated that rigid or flexible, permanent or absorbable prosthetic materials do not affect perioperative pulmonary or infectious complications; only resected rib number and concomitant lung resection independently predict pulmonary morbidity (21).

Three-dimensional (3D) printing technology has demonstrated significant potential in the application of titanium alloys, particularly in the context of chest wall reconstruction. This technology enables the production of customizable components that can be precisely tailored to the unique anatomical requirements of individual patients, ensuring an accurate fit with the affected area. The primary advantage of 3D printing lies in its capacity to fabricate complex geometries and structures that cater to the diverse physiological needs of patients. Furthermore, titanium alloys offer excellent biocompatibility and sufficient mechanical strength, do not interfere with postoperative imaging assessments, and can be engineered to include movable chest joints, among other benefits (22, 23). Clinically, it is primarily utilized for patients with multiple rib fractures on both sides or complete sternal defects. Nevertheless, its relatively high cost, lengthy preoperative preparation period, and the inability to make intraoperative modifications constrain its broader adoption. In this study, titanium alloy components were used to repair thoracic defects in 7 patients, representing 18.42% of the cohort. Postoperatively, there were no instances of displacement, breakage, or exposure of the implanted components. Vanstraelen et al. conducted a matched analysis and confirmed that biologic and synthetic prostheses present similar rates of surgical site complications requiring reoperation; longer operative time and greater blood loss increase complication risk (24).

Extensive soft tissue defects of the chest wall necessitate flap repair, particularly when synthetic materials are employed in the reconstruction of the chest wall. It is imperative to utilize soft tissue with a robust blood supply to cover the defect, thereby preventing the exposure of the repair material and mitigating severe complications. In this study, the 42 patients with substantial soft tissue defects were not amenable to direct suturing. In both domestic and international literature, the latissimus dorsi flap is extensively utilized in chest wall reconstruction surgeries due to its numerous advantageous characteristics. Firstly, the latissimus dorsi flap is characterized by a highly reliable blood supply, ensuring adequate perfusion during both the surgical procedure and the postoperative healing phase. This enhances the likelihood of surgical success and optimizes healing outcomes. Secondly, the flap is distinguished by a relatively long vascular pedicle, facilitating greater flexibility in its rotation and positioning during surgery (20). Furthermore, the latissimus dorsi flap exhibits a considerable range of motion, facilitating its use in covering extensive defect areas. This characteristic is particularly advantageous in scenarios requiring large-scale tissue repair, where the flap demonstrates exceptional efficacy. The latissimus dorsi flap can provide substantial tissue coverage, serving not only to cover defects but also to partially restore the function and aesthetic appearance of the chest wall. These attributes render the latissimus dorsi flap a critical option in chest wall reconstruction, especially in addressing complex and extensive defects, where its application proves particularly effective (25–27). This adaptability allows it to accommodate various defect locations and configurations effectively. In clinical practice, the majority of cases we encounter involve patients with chest wall recurrence or radiation-induced ulcers following breast cancer surgery. The tissue surrounding these lesions is often compromised. The axillary clearance and subsequent axillary radiotherapy associated with modified radical mastectomy for breast cancer increase the likelihood of thoracodorsal vascular damage, complicating vascular pedicle dissection. Although the latissimus dorsi muscle offers extensive tissue coverage, its use is limited due to the necessity of repositioning the patient during surgery, potential donor site morbidity, and the requirement for skin grafting. Consequently, the pedicled latissimus dorsi flap is infrequently selected as the primary option for chest wall defect repair. Instead, the deep inferior epigastric artery perforator (DIEP) flap and the transverse rectus abdominis myocutaneous (TRAM) flap are predominantly utilized in our clinical practice. These flaps are favored due to their reliable vascular supply, substantial tissue provision, minimal donor site morbidity, and the advantage of not requiring intraoperative repositioning. Due to the impact of tumor infiltration, radiotherapy, inflammation, and infection on the blood vessels at the recipient site, as well as other limiting conditions, it is typically challenging to provide two sets of recipient site blood vessels for anastomosis. Ensuring a reliable blood supply to the flap during surgery is crucial. To address this, we designed the abdominal flap as a Deep Inferior Epigastric Perforator (DIEP) flap requiring anastomosis on one side, and a pedicled Transverse Rectus Abdominis Myocutaneous (TRAM) flap on the opposite side, thereby ensuring two sets of reliable blood supplies for the entire flap. Consequently, the incidence of postoperative complications associated with flap blood supply or vascular crisis is notably low, at 2.38%.

Vanstraelen et al. further showed that microvascular free flaps (MVFF) cover larger defects, achieve higher R0 resection rates and lower local recurrence, with postoperative complications comparable to pedicled flaps (28). Giordano et al. reported that acellular dermal matrix (ADM) yields significantly fewer surgical-site complications than synthetic mesh in oncologic chest wall reconstruction, supporting selective use of biologic materials in high-risk patients (29). The incidence of postoperative complications following thoracic reconstruction is notably high, with reported rates between 24% and 46% (30, 31). Among these, systemic postoperative complications are particularly prevalent, encompassing postoperative bleeding, atrial fibrillation, deep vein thrombosis, and respiratory failure (32). These complications adversely impact patients’ postoperative recovery and may result in severe health issues, potentially posing life-threatening risks. Postoperative hemorrhage represents a frequent complication following thoracic reconstruction, potentially attributable to vascular injury incurred during the surgical procedure. Atrial fibrillation, another prevalent cardiac complication, may be associated with surgical stress and an elevated cardiac workload (30). Deep vein thrombosis can arise due to diminished postoperative mobility and impaired blood circulation (32). Furthermore, respiratory failure constitutes a severe complication post-thoracic surgery, potentially resulting from postoperative pulmonary infection, atelectasis, or other respiratory issues. To mitigate the occurrence of these complications, it is crucial to conduct comprehensive preoperative risk assessments and implement meticulous postoperative monitoring strategies. By undertaking thorough preoperative evaluations, patients at elevated risk can be identified, allowing for the formulation of tailored postoperative management plans aimed at minimizing the incidence of complications (33–35). In our clinical practice, the most frequently observed complications are poor wound healing in the affected area (30.95%) and complications related to pulmonary insufficiency, including hypoxemia, pleural effusion, and atelectasis (11.9%). This may be attributed to the fact that the study population predominantly comprises patients with chest wall radiation ulcers.

## 5 Conclusion

The clinical management of primary or recurrent malignant chest wall tumors and radiation-induced ulcers is extremely challenging, requiring a multidisciplinary collaborative diagnosis and treatment approach to optimize surgical planning. Following surgical resection of such lesions, extensive full-thickness chest wall defects are often incurred, necessitating simultaneous reconstruction of the bony thoracic cage and surrounding soft tissues. Therefore, the selection of appropriate repair materials and surgical techniques is of paramount importance. Ideal chest wall repair must meet five core criteria: complete resection of the lesion, reconstruction of the bony supporting structure of the thoracic cage, full coverage of soft tissues, ensuring flap survival without postoperative respiratory dysfunction, and consideration of certain aesthetic outcomes.

This study confirms that the combination of bone cement with mesh and titanium alloy with mesh are effective strategies for repairing large-area chest wall defects, which can provide stable bony support and promote soft tissue healing. In addition, the combined application of free deep inferior epigastric artery perforator (DIEP) flaps and transverse rectus abdominis myocutaneous (TRAM) flaps not only achieves extensive soft tissue coverage but also preserves the vascular supply and functional integrity of the donor site. This combined flap repair strategy exhibits significant advantages in the repair of chest wall defects, particularly radiation-induced ulcers, with definite clinical efficacy, and thus is worthy of further promotion and application in relevant surgical procedures.

## Data Availability

The original contributions presented in the study are included in the article/Supplementary Material, further inquiries can be directed to the corresponding author.

## References

[1] Scarnecchia E Liparulo V Capozzi R Ceccarelli S Puma F Vannucci J. Chest wall resection and reconstruction for tumors: analysis of oncological and functional outcome. J Thorac Dis. (2018) 10:S1855–63. 10.21037/jtd.2018.05.191 30026972 PMC6035939

[2] Viani GA Gouveia AG Louie AV Korzeniowski M Pavoni JF Hamamura AC Stereotactic body radiotherapy to treat breast cancer oligometastases: a systematic review with meta-analysis. Radiother Oncol. (2021) 164:245–50. 10.1016/j.radonc.2021.09.031 34624408

[3] Matsumine H Kirita M Sakurai H. Reconstruction with a 180-degree rotationally divided Latissimus-dorsi-musculocutaneous flap after the removal of locally advanced breast cancer. Plast Reconstr Surg Glob OPEN. (2014) 2:e217. 10.1097/GOX.0000000000000190 25426400 PMC4229276

[4] Liu J Han J Ji G Zhang T Xie S Liu Y Laparoscopic harvest and free transplantation of great omentum flap for extensive tissue defects in complex wounds. JPRAS Open. (2024) 39:1–10. 10.1016/j.jpra.2023.10.012 38076652 PMC10700857

[5] Li C Lv H Du Y Zhu W Yang W Wang X Biologically modified implantation as therapeutic bioabsorbable materials for bone defect repair. Regen Ther. (2022) 19:9–23. 10.1016/j.reth.2021.12.004 35024389 PMC8732753

[6] Wüster J Neckel N Sterzik F Xiang-Tischhauser L Barnewitz D Genzel A Effect of a synthetic hydroxyapatite-based bone grafting material compared to established bone substitute materials on regeneration of critical-size bone defects in the ovine scapula. Regen Biomater. (2024) 11:rbae041. 10.1093/rb/rbae041 38903563 PMC11187503

[7] Ying D Zhang T Qi M Han B Dong B. Artificial bone materials for infected bone defects: advances in antimicrobial functions. ACS Biomater Sci Eng. (2025) 11:2008–36. 10.1021/acsbiomaterials.4c01940 40085817

[8] Zhang T Zhou W Yang W Bi J Li H Gao X Vancomycin-encapsulated hydrogel loaded microarc-oxidized 3D-printed porous Ti6Al4 V implant for infected bone defects: reconstruction, anti-infection, and osseointegration. Bioact Mater. (2024) 42:18–31. 10.1016/j.bioactmat.2024.07.035 39262845 PMC11388676

[9] Zhang H Qiao W Liu Y Yao X Zhai Y Du L. Addressing the challenges of infectious bone defects: a review of recent advances in bifunctional biomaterials. J Nanobiotechnology. (2025) 23:257. 10.1186/s12951-025-03295-0 40158189 PMC11954225

[10] Jo GY Ki SH. Analysis of the chest wall reconstruction methods after malignant tumor resection. Arch Plast Surg. (2023) 50:010–6. 10.1055/s-0042-1760290 PMC9902099 36755660

[11] Fang L Chen YJ Wu GY Zou QY Wang ZG Zhu G Ribs formed by prolene mesh, bone cement, and muscle flaps successfully repair chest abdominal wall defects after tumor resection: a long-term study. Chin Med J (Engl). (2017) 130(12):1510–1. 10.4103/0366-6999.207473 28584220 PMC5463487

[12] Fracol M Grim M Lanier ST Fine NA. Vertical skin paddle orientation for the Latissimus dorsi flap in breast reconstruction: a modification to simultaneously correct Inferior pole constriction and improve projection. Plast Reconstr Surg. (2018) 141:598–601. 10.1097/PRS.0000000000004103 29135896

[13] Khoobehi K. Invited discussion on: safety of large-volume immediate fat grafting for Latissimus dorsi-only breast reconstruction: results and related complications in 95 consecutive cases. Aesthetic Plast Surg. (2020) 45:76–7. 10.1007/s00266-020-01959-w 32995983

[14] Bodin F Dissaux C Steib J-P Massard G. Complex posterior thoracic wall reconstruction using a crossover combined latissimus dorsi and serratus anterior free flap. Eur J Cardiothorac Surg. (2015) 49:1008–9. 10.1093/ejcts/ezv141 25825263

[15] Groth AK Pazio ALB Kusano LDC Lupion F Itikawa WM Legnani BC Thoracic wall reconstruction: surgical planning in extended malignant resections. Ann Plast Surg. (2020) 85:531–8. 10.1097/SAP.0000000000002298 32079809

[16] Muguruza I Aranda JL García-Yuste M. Treatment of lung cancer with chest wall invasion. Arch Bronconeumol. (2011) 47:27–32. 10.1016/S0300-2896(11)70008-4 21300215

[17] Isaac KV Elzinga K Buchel EW. The best of chest wall reconstruction: principles and clinical application for Complex oncologic and sternal defects. Plast Reconstr Surg. (2022) 149:547e–62e. 10.1097/PRS.0000000000008882 35196698

[18] Deschamps C Tirnaksiz BM Darbandi R Trastek VF Allen MS Miller DL Early and long-term results of prosthetic chest wall reconstruction. J Thorac Cardiovasc Surg. (1999) 117:588–92. 10.1016/S0022-5223(99)70339-9 10047664

[19] Ng CSH. Recent and future developments in chest wall reconstruction. Semin Thorac Cardiovasc Surg 2015:27:234–9. 10.1053/j.semtcvs.2015.05.002 26686454

[20] Palmesano M Lisa A Storti G Bottoni M Gottardi A Colombo G Resection to restoration: assessing the synergy of polypropylene mesh (marlex®) combined with methyl-methacrylate and latissimus dorsi flap for primary chest wall sarcomas. J Plast Reconstr Aesthet Surg. (2024) 93:157–62. 10.1016/j.bjps.2024.04.022 38691953

[21] Spicer JD Shewale JB Antonoff MB Correa AM Hofstetter WB Rice DC The influence of reconstructive technique on perioperative pulmonary and infectious outcomes following chest wall resection. Ann Thorac Surg. (2016) 102:1653–9. 10.1016/j.athoracsur.2016.05.072 27526650

[22] Attarilar S Ebrahimi M Djavanroodi F Fu Y Wang L Yang J. 3D Printing technologies in metallic implants: a thematic review on the techniques and procedures. Int J Bioprinting. (2024) 7:306. 10.18063/ijb.v7i1.306 PMC7875061 33585711

[23] Ren B Wan Y Liu C Wang H Yu M Zhang X Improved osseointegration of 3D printed ti-6Al-4 V implant with a hierarchical micro/nano surface topography: an *in vitro* and *in vivo* study. Mater Sci Eng C. (2021) 118:111505. 10.1016/j.msec.2020.111505 33255064

[24] Vanstraelen S Bains MS Dycoco J Adusumilli PS Bott MJ Downey RJ Biologic versus synthetic prosthesis for chest wall reconstruction: a matched analysis. Eur J Cardio-Thorac Surg Off J Eur Assoc Cardio-Thorac Surg. (2023) 64:ezad348. 10.1093/ejcts/ezad348 PMC11032705 37846030

[25] Ferraro P Cugno S Liberman M Danino MA Harris PG. Principles of chest wall resection and reconstruction. Thorac Surg Clin. (2010) 20:465–73. 10.1016/j.thorsurg.2010.07.008 20974430

[26] Bakri K Mardini S Evans KK Carlsen BT Arnold PG. Workhorse flaps in chest wall reconstruction: the pectoralis major, latissimus dorsi, and rectus abdominis flaps. Semin Plast Surg. (2011) 25:043–54. 10.1055/s-0031-1275170 PMC3140231 22294942

[27] Sood R Easow JM Konopka G Panthaki ZJ. Latissimus dorsi flap in breast reconstruction: recent innovations in the workhorse flap. Cancer Control. (2018) 25:1073274817744638. 10.1177/1073274817744638 29334788 PMC5933575

[28] Vanstraelen S Ali B Bains MS Shahzad F Allen RJ Matros E The contribution of microvascular free flaps and pedicled flaps to successful chest wall surgery. J Thorac Cardiovasc Surg. (2023) 166:1262–1272.e2. 10.1016/j.jtcvs.2023.05.018 37236598 PMC10528168

[29] Giordano S Garvey PB Clemens MW Baumann DP Selber JC Rice DC Synthetic mesh versus acellular dermal matrix for oncologic chest wall reconstruction: a comparative analysis. Ann Surg Oncol. (2020) 27:3009–17. 10.1245/s10434-019-08168-z 32152778

[30] Chung MM Pan C Hayashi H Kandula V Zhao Y Levine D Significance of isolated postoperative atrial fibrillation in thoracic aortic aneurysm repair. J Thorac Cardiovasc Surg. (2025) 169:617–626.e7. 10.1016/j.jtcvs.2023.12.023 38191071

[31] Duranti L Tavecchio L. New perspectives in prosthetic reconstruction in chest wall resection. Updates Surg. (2023) 75:1093–102. 10.1007/s13304-023-01562-z 37402065

[32] Feng B Lin J Jin J Qian W-W Wang W Weng X-S. Thirty-day postoperative complications following primary total knee arthroplasty: a retrospective study of incidence and risk factors at a single center in China. Chin Med J (Engl). (2017) 130:2551–6. 10.4103/0366-6999.213071 28836570 PMC5678253

[33] Chandler D Mosieri C Kallurkar A Pham AD Okada LK Kaye RJ Perioperative strategies for the reduction of postoperative pulmonary complications. Best Pract Res Clin Anaesthesiol. (2020) 34:153–66. 10.1016/j.bpa.2020.04.011 32711826

[34] Lans TE Van Der Pol C Wouters MW Schmitz PIM Van Geel AN. Complications in wound healing after chest wall resection in cancer patients; a multivariate analysis of 220 patients. J Thorac Oncol. (2009) 4:639–43. 10.1097/JTO.0b013e31819d18c9 19357542

[35] Yang H Tantai J Zhao H. Clinical experience with titanium mesh in reconstruction of massive chest wall defects following oncological resection. J Thorac Dis. (2015) 7(7):1227–1234. 10.3978/j.issn.2072-1439.2015.05.13 26380739 PMC4522483

